# Jellytoring: Real-Time Jellyfish Monitoring Based on Deep Learning Object Detection

**DOI:** 10.3390/s20061708

**Published:** 2020-03-19

**Authors:** Miguel Martin-Abadal, Ana Ruiz-Frau, Hilmar Hinz, Yolanda Gonzalez-Cid

**Affiliations:** 1 Department of Mathematics and Computer Science, Systems Robotics and Vision Group (SRV), Universitat de les Illes Balears, 07122 Palma, Spain; yolanda.gonzalez@uib.es; 2 Department of Marine Ecosystem Dynamics, IMEDEA (CSIC-UIB), Institut Mediterrani d’Estudis Avançats, 07190 Esporles, Spain; anaruiz@imedea.uib-csic.es (A.R.-F.); hhinz@imedea.uib-csic.es (H.H.)

**Keywords:** deep learning, object detection, jellyfish quantification, jellyfish monitoring

## Abstract

During the past decades, the composition and distribution of marine species have changed due to multiple anthropogenic pressures. Monitoring these changes in a cost-effective manner is of high relevance to assess the environmental status and evaluate the effectiveness of management measures. In particular, recent studies point to a rise of jellyfish populations on a global scale, negatively affecting diverse marine sectors like commercial fishing or the tourism industry. Past monitoring efforts using underwater video observations tended to be time-consuming and costly due to human-based data processing. In this paper, we present Jellytoring, a system to automatically detect and quantify different species of jellyfish based on a deep object detection neural network, allowing us to automatically record jellyfish presence during long periods of time. Jellytoring demonstrates outstanding performance on the jellyfish detection task, reaching an *F*1 *score* of 95.2%; and also on the jellyfish quantification task, as it correctly quantifies the number and class of jellyfish on a real-time processed video sequence up to a 93.8% of its duration. The results of this study are encouraging and provide the means towards a efficient way to monitor jellyfish, which can be used for the development of a jellyfish early-warning system, providing highly valuable information for marine biologists and contributing to the reduction of jellyfish impacts on humans.

## 1. Introduction

During the past decades, the marine environment has been under increased pressure by human activities, such as the over-exploitation of marine species [1], the destruction and modifications of habitats [2], the introduction of alien species [3], as well as pollution [4] and human-induced climate change [5,6]. These pressures have caused highly relevant changes in the composition and distribution of marine organisms [7].

The detection and quantification of changes in marine species are of vital importance to monitor environmental status and its change over time, in particular, the benefits society derives from ecosystems, known as ecosystem services [8]. Furthermore, the capacity to monitor is critical in the assessment of the effectiveness of control or recovery measures implemented through management.

Visual observations of marine organisms using video cameras are increasingly adopted to monitor the marine environment due to the low cost of this technology and the wide applicability within a challenging environment for humans. Until recently, video observations have been processed and classified by human observers, which in many instances is time-consuming and consequently financially costly [9,10].

In addition, the underwater environment is a highly dynamic environment, where a wide range of variables such as water turbidity, scale deformations, illumination variations, presence of flares, color distortions or light can affect the quality of the images collected, making data extraction a challenging undertaking.

Over the last decade, automatic detection methods have arisen as a cost-effective way for image location and classification [11], this is highly relevant in regards to the increasing amount of image data that is being collected from the marine environment. In general, images of animal species are used to record and quantify their density, distribution and behavior [12,13,14,15]. Getting to determine where objects are located in a given image (object localization) and which category each object belongs to (object classification) can be useful in a multitude of scenarios and implemented for multiple applications. In the marine environment object detection and classification has been used among others to record fish presence and recognition [16,17,18,19], to monitor marine turtles [20] or in the classification of planktonic organisms [21].

General existing solutions for organisms automatic detection can be roughly classified into two groups: traditional computer vision algorithms or artificial intelligence based approaches.

Traditional computer vision algorithms use feature detection algorithms (SIFT, SURF, BRIEF, etc.) to extract feature information from the image (position of corners, edges, blobs, etc.). An object is recognized in a new image by individually comparing its features to a database and finding candidate matching features. The difficulty with these traditional approaches is the necessity to choose which features are important for each task. As the number of organisms to classify increases, feature extraction becomes more complex [22].

Artificial intelligence approaches, in turn, can be divided into two groups, machine learning and deep learning approaches:

Machine learning based approaches perform an informative region selection followed by a feature extraction of the selected regions (e.g., SIFT [23], HOG [24], Haar-like [25]) and finally a region classification using a determined method (e.g., Supported Vector Machine [26], AdaBoost [27], Deformable Part-based Model [28]). Still, the feature extraction process needs to be determined manually.

Deep learning based frameworks for image processing and object detection specifically, mostly rely on region-based *Convolutional Neural Networks* (CNN) like R-CNN [29] or its performance evolutions: Fast R-CNN [30] and Faster R-CNN [31], to generate deeper neural networks with more layers, able to learn and extract more complex features. Here, the full process is automated, with no need of a previous feature extraction, as the network inputs an image and is able to extract its own features.

In this paper, we present Jellytoring, a system to automatically detect and quantify different species of jellyfish based on a deep object detection neural network. Within the context of human–environment interactions, jellyfish are organisms that can create a multitude of impacts on human wellbeing. Among others, the presence of jellyfish aggregations can clog seawater intake screens in water desalination and power plants, causing power reductions and shutdowns [32,33], leaving entire populations without electrical supply. In aquaculture, large aggregations of jellyfish can cause important socio-economic impacts by killing farmed fish in pens [34,35]. In commercial fishing, jellyfish can interfere with fishing operations by constituting a health hazard to fishermen when retrieving the nets, by splitting the fishing nets due to the weight of the jellyfish in the nets or by ruining the catch [36]. Additionally, jellyfish are known to create negative impacts on coastal tourism by generating unpleasant experiences among coastal users with associated impacts on tourism revenues and the tourism industry [37].

The development of an automatic jellyfish detection and identification system could contribute to the reduction of jellyfish impacts on humans, providing the means towards an effective acquisition of jellyfish presence surveillance data which could be used for the development of a jellyfish early-warning system. The nature and characteristics of jellyfish, however, are challenging aspects to overcome in the development of such a system. Jellyfish are often translucent organisms whose bodies can adopt significantly different configurations, due to the movement of their tentacles in relation to their main body structure, i.e., the bell or umbrella. These aspects, translucent nature and changing shapes, together with the added difficulties of object detection in underwater environments, represent challenging conditions for the development of a jellyfish detection system.

We focused on the North-Western Mediterranean sea, an area with a high human population and a popular tourism destination, where human–jellyfish interactions are frequent. Specifically, we studied three jellyfish species which are common during the summer months and which often cause undesired effects on tourism satisfaction, namely *Pelagia noctiluca*, *Cotylorhiza tuberculata* and *Rhizostoma pulmo*.

The remainder of this paper is structured as follows. Section 2 reviews related work on jellyfish detection, quantification and monitoring and highlights our main contributions. Section 3 describes the used neural network architecture and its training details. Section 4 describes the adopted methodology and materials used in this study. The experimental results are presented in Section 5. Finally, Section 6 presents the main conclusions and outlines future work.

## 2. Related Work and Contributions

This section briefly describes the existing related efforts on jellyfish detection and monitoring. The main contributions of this paper are highlighted at the end of this section.

### 2.1. State-of-the-Art

During the last decades, the monitoring of jellyfish species has mostly been carried out manually, relying on human visual observations to detect, identify and quantify specimens; that is either by direct observations made in the field [38] or by using video recordings that subsequently needed manual analysis [39]. The use of aerial vehicles has also been adopted, to cover a larger study areas [40]. However in general, visual observations tend to be slow, labor and resource intensive, thus restricting the spatial and temporal extent of the studies [41,42].

Some studies have used the aid of traditional computer vision techniques to automate the detection of jellyfish. Rife et al. [43] tested various image filtering techniques and segmentation algorithms to track deep-ocean jellyfish on conventional camera imagery. However, this implementation only considers a generic jellyfish class, not distinguishing between different species. Moreover, the selected combination of filtering and segmentation algorithm does not allow for a real-time tracking application.

As in many other research areas, the recent development of deep learning architectures has offered major improvements in accuracy for observational ecological studies [44], dealing at the same time with the spatial and temporal limitations of human visual observation [45]. Even so, the application of deep learning for jellyfish detection has been very limited. To our knowledge, only two peer-reviewed publications have focused on the subject.

Kim et al. [46] make use of an unspecified CNN along with collaborative filters to build a jellyfish recognition algorithm for sea surface imagery taken by an unmanned aerial vehicle. Similar to the studies mentioned above, this study does also not distinguish between different species of jellyfish. Furthermore, limiting image capture to the water surface underestimates jellyfish numbers, as jellyfish distribution is not limited to surface waters only and tend to occupy a large extent of the underlying water column.

French et al. [47] implement a 10-layer VGG-style CNN architecture to detect jellyfish in underwater sonar imagery, correctly classifying up to a 90% of the jellyfish for the test set. The use of sonar imagery presents some advantages, like the usability at deeper areas where light does not reach. On the other hand, it suffers from some drawbacks versus the usage of normal camera imagery, like lower resolution or grey-scale coloring, complicating the detection task. This study did not differentiate between different jellyfish species.

Finally, we found that none of these works performed a jellyfish quantification to provide information of occurrences over time, nor used time series processing techniques to improve the detection rate that allowed for the implementation of a monitoring algorithm.

### 2.2. Main Contributions

The main contributions of this paper are composed of:

1. A real-time jellyfish monitoring system based on deep learning object detection named Jellytoring, which provides highly valuable information to biologists, ecologists and conservationists on the presence and occurrence of different species of jellyfish in an studied area.
2. The usage of a deep CNN, trained several times to fine tune its hyperparameters to detect and classify up to three different species of jellyfish on underwater images. We evaluated the network on a test set of images, comparing its results to other neural networks.
3. First system to achieve real-time automatic quantification and identification of different species of jellyfish. We designed and tested an algorithm that can be executed in real-time and uses the network detection to quantify and monitor jellyfish presence on video sequences.
4. The creation of a publicly available dataset used for the training and testing of the neural network and the quantification algorithm, containing the original images and corresponding annotations.

## 3. Deep Learning Approach

This section describes the framework and network selection process along with its architecture and training details.

### 3.1. Framework and Network Selection

There are several deep learning frameworks based on CNN that can be used to extract instance information from images. They go from the standard region proposal based object detection frameworks of Faster R-CNN [31] or some of its direct evolutions like FPN [48], mask R-CNN [49] or RFCN [50]; to regression-based ones like YOLO [51] or SSD [52]; or even more specific frameworks like deep salient object detection [53].

In our case, we aim to implement an object detection framework able to detect and classify up to three species of jellyfishes present in underwater images, with no need of obtaining the pixel-wise segmentation of the detected instances nor any extra feature that could slow the process. We wanted to ensure that the system is able to perform real-time quantification on a wide spectrum of setups, widening its applicability.

Taking into account both the computational cost along with the features of the aforementioned frameworks and the requirements of our application, we opted for the usage of the Faster R-CNN framework. This framework allow us to obtain the jellyfish instances bounding boxes and its classification, while balancing the detection performance and computational cost trade off by selecting an adequate deep learning architecture for this specific task.

Due to the slow movement of the jellyfish, an architecture with high detection performance, despite having a relatively high image analysis time is suitable. Therefore, based on the performance metrics provided by Google on tests [54] conducted for diverse object detection architectures over the COCO dataset [55], we selected the Faster R-CNN-based implementation of the Inception ResNet v2 [56] architecture. It uses a region proposal network to generate object position instances and then the Inception ResNet v2 to fine-tune these proposals and output a final prediction, presenting a two-stage detection framework.

Inception ResNet v2 is a very deep CNN with over 450 layers that can efficiently learn to identify objects in images, outputting instance bounding boxes and classifying them into one of the specified classes with a confidence percentage.

Selecting appropriate kernel sizes for the convolutional layers is a crucial aspect when detecting objects in an image, as the same object may show variations in shape and size. Larger kernels are preferred for the detection of bigger objects while smaller kernels are favored for smaller ones. To address this variation, Inception-ResNet V2 architecture performs multiple parallel convolutions using different kernel sizes, making the network “wider” rather than “deeper”. The blocks of layers containing these convolutions are called Inception Modules [57].

**Figure 1:**
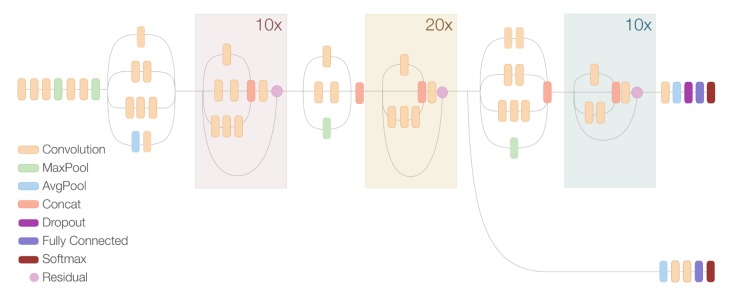
Inception ResNet v2 architecture. Credit: Google AI Blog.

The network also uses Residual Connections [58], through which the output of the convolution operation of the Inception Module is added to the input. This introduces shortcuts in the model resulting in more optimal and accurate networks. This architecture combines Inception Modules and Residual Connections which results in the Inception-ResNet modules. Figure 1 shows a compressed view of the whole Inception ResNet v2 architecture. More in-depth information about this architecture can be found in [56].

### 3.2. Training Details

The Inception-ResNet V2 architecture is trained by means of readjusting the kernel values in the convolutional layer filters, back-propagating the loss computed over the predictions obtained on the softmax layers.

Due to the high number of layers, the loss becomes small and insufficient to update the kernel values properly. To prevent the middle part of the network from “dying out” during the backpropagation process, an auxiliary classifier is applied at the output of the second block of Inception-ResNet modules. In this way, an auxiliary loss is computed and added to the prior one as shown in Equation (1).

(1) Total_loss=main_loss+aux_loss×0.3.

To train the network and adjust the kernel weights, the smooth L1 location backpropagation loss function is used, which loss increases as the predicted bounding box location diverges from the ground truth. Additionally, the Momentum optimizer algorithm together with gradient clipping strategies [59] are utilized to achieve a minimum global error.

The architecture used for this application had already been trained over the COCO dataset [55]. To retrain the network, it is needed a set of images containing different species of jellyfish and their corresponding ground truth annotations, where the position and class of each jellyfish instance are indicated.

## 4. Methodology

This section introduces the general workflow of Jellytoring and subsequently provides details of each work step taken i.e., the acquisition and labeling of the data from the training and testing sets, the tested network hyperparameters and studied combinations, the validation process and evaluation metrics and finally, the quantification algorithm.

### 4.1. Workflow

First, a set of images containing jellyfish needs to be forwarded into a frozen version of a trained model of the deep object detection neural network. After its inference, the network generates the jellyfish detection.

Following, this detection is optimized by a *non-maxima suppression* (nms) algorithm [60], deleting overlapping ones. Then, the final predictions for each analyzed image are obtained by deleting instances with an associated confidence lower than a selected threshold value ($C_{thr1}$). These predictions can be used to measure jellyfish occurrences and species recognition in the forwarded images on its own.

Furthermore, if the initial source of data is a video sequence, the network detection can be forwarded into the quantification algorithm to obtain the evolution of number and species of jellyfish present on the video sequence. This algorithm first deletes instances with an associated confidence lower than a selected threshold value ($C_{thr2}$) and then applies time series processing techniques. More in depth information about the quantification algorithm is provided in Section 4.6.

**Figure 2:**
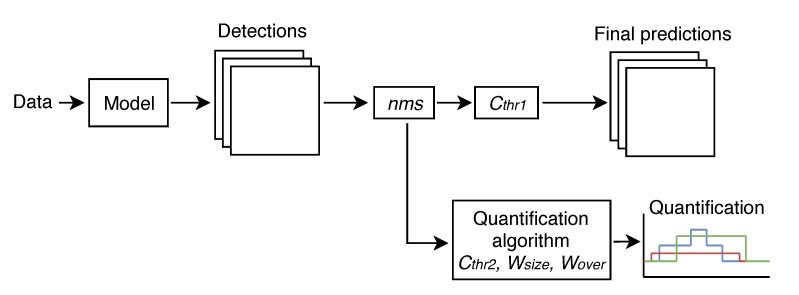
Jellytoring workflow.

Figure 2 represents the workflow of Jellytoring.

### 4.2. Data Collection

The present study focuses on three jellyfish species, namely *Pelagia noctiluca*, *Rhizostoma pulmo* and *Cothyloriza tuberculata*. To obtain the needed data to train and test the neural network, we extracted images containing instances of the studied jellyfish from underwater video recordings.

The first source of data consisted of a series of recordings we generated by mounting a GoPro camera onto a platform and deploying it at the seafloor, pointing upwards. In order to obtain a variety of exposure conditions, recordings were done during different times of the day, over different seabed types and weather conditions. Using this method we generated up to 4 h of recordings. Secondly, to obtain additional data, we examined diverse social media sites publicly available videos where appeared instances of the three studied jellyfish. From these sources, we extracted a total of 842 images, each one containing at least one jellyfish instance. When possible, images containing more than one instance were extracted. The resolution of the images range from 320×240 to 1920×1080 pixels, they can be forwarded into the network without any processing, as the network is able to process different image and bounding boxes sizes thanks to its multiple feature extraction kernels sizes and shapes.

**Figure 3:**
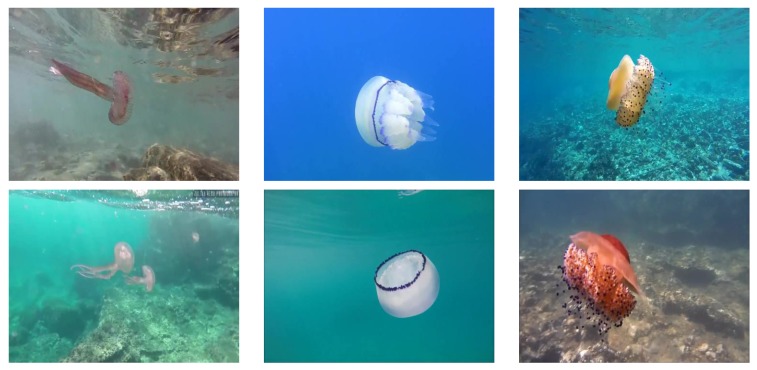
Images from the dataset showing the three jellyfish species under different environmental conditions. **Left**: *P. noctiluca*, **center**: *R. pulmo*, **right**: *C. tuberculata*.

We built a varied dataset containing jellyfish instances under different conditions, such as jellyfish coloration, position and size; or water illumination, depth and turbidity. We obtained a varied and robust dataset to train the neural network without overfitting the training data and to test it on different scenarios to ensure its wide usability. Figure 3 shows sample images from the dataset show-casing different environmental conditions.

**Figure 4:**
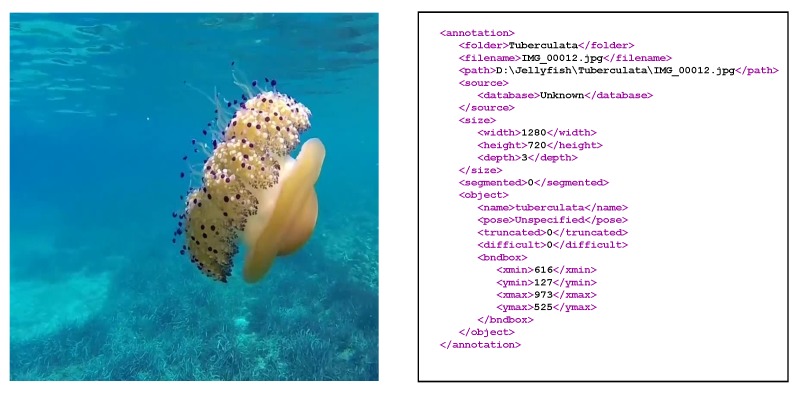
**Left**: Original image. **Right**: Corresponding ground truth “.xml” file, specifying the jellyfish location and class.

To log the presence of the different jellyfish species, annotation files were generated using the LabelImg tool [61]. For each image, a bounding box around each jellyfish instance was drawn and was classified according to its species. The LabelImg tool then generates an “.xml” file containing the position and classification of each instance within the corresponding image. A total of 962 jellyfish occurrences were recorded, 327 corresponding to *Pelagia noctiluca*, 292 to *Rhizostoma pulmo* and 343 to *Cothyloriza tuberculata*. Figure 4 shows an original image along with its ground truth “.xml” text file.

### 4.3. Hyperparameter Selection

When training a neural network the value of specific hyperparameters can determine some of the network features and the training process itself. To find the values of these hyperparameters that offer the best performance, the network was trained using different values and combinations.

The considered hyperparameters were:

- Data augmentation: it is a technique that consists of applying random rotations and horizontal and vertical transformations to the training images in order to train over more diverse data, helping to reduce overfitting [62].
- Learning rate: this hyperparameter modifies the training step size the network uses when searching for an optimal solution. We also studied the effect of applying a decay learning rate, which consists of lowering the learning rate value as the training progresses [63].
- Number of iterations: this hyperparameter sets the number of times the network back-propagates and trains [63].

Table 1 shows the values and combinations of hyperparameters that we used to train the neural network.

### 4.4. Validation

We conducted twelve different experiments, each one assessing the performance of hyperparameter combination. When training the network, we made use of the 10k-fold cross-validation method [64]. Through this method, the dataset is split into ten equally sized subsets and the network is trained ten times, each time using two different subsets as the test data (20% of the dataset) and the remaining eight subsets as training data (80% of the dataset). This method reduces the variability of the results, as these are less dependent on the selected test and training datasets, therefore obtaining a more accurate performance estimation.

Using the 10k-fold cross-validation, ten models were generated for each experiment, $M_{K}^{i}$, where *K* = 1...12 represents the experiment number and *i* = 1...10 the model index. We ran the ten output models with their corresponding test subsets, obtaining jellyfish detection of all the models.

To remove overlapped detection and obtain the final predictions of each model, $P_{K}^{i}$, an nms algorithm is applied. This algorithm computes the intersection area between detection and eliminates the least confident ones when the intersection area is greater than a threshold. Threshold values for this type of application are usually set between 30–70% [65,66], in our case, we selected a fairly restrictive threshold of 40%, as it is not common that two or more jellyfish appear superimposed in the images.

**Figure 5:**
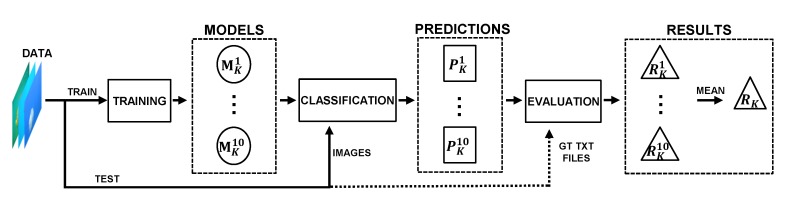
Experiment *K* validation process. For each one of the twelve hyperparameter combinations, the network was trained ten times using the k-fold cross-validation method, outputting ten models. These models were run and evaluated over the test data. Finally, the results of the models were obtained and its mean performance calculated.

From these predictions, each model is evaluated in terms of detection performance, obtaining its results metrics $R_{K}^{i}$. Finally, the detection performance $R_{K}$ of each experiment is computed as the mean of its ten $R_{K}^{i}$ models performance. The best model *M* corresponding to the experiment that presented the best results is selected to generate the quantification and monitoring predictions. The validation process of the experiments is shown in Figure 5.

### 4.5. Model Evaluation

The first step to evaluate a model and measure its performance is to classify each one of the predictions over the test set data as either correct (*True Positive*, TP) or incorrect (*False Positive*, FP). To do so, we used the *Intersection over Union* (IoU) measure, which provides the similarity between the predicted and the ground-truth bounding-boxes areas. The IoU value is defined as the area of the intersection between bounding-boxes divided by the union of the bounding-boxes areas (Equation (2)).

(2) $$IoU=\frac{A_{intersection}}{A_{union}}.$$

To determine whether a prediction is a TP or an FP, an IoU threshold value needs to be established. Following the criteria applied in the PASCAL VOC challenge [67], this threshold was set at *thr_iou_* = 0.5. A prediction is classified as TP if the IoU value with any ground truth bounding-box is greater than the *thr_iou_* and the predicted class matches the corresponding one specified in the ground truth box. Otherwise, the prediction is classified as an FP (Equation (3)).

(3) $$Prediction=\left\{\begin{array}{cc} TP, & \mathrm{if}\;IoU>=thr_{iou}\;\&{\mathrm{Class}}_{\mathrm{pred}}={\mathrm{Class}}_{\mathrm{gt}},\\ FP, & \mathrm{otherwise}. \end{array}\right.$$

Finally, ground-truth instances that do not have a IoU > thr_iou_ with any prediction are counted as undetected instances (*False Negatives*, FN).

Once each prediction is classified as either TP or FP, and the number of FN is obtained, evaluation metrics are computed.

*Average Precision* (AP) [68] is one the most frequently used metrics in object detection applications. It is largely used in object detection competitions such as PASCAL VOC [67], ImageNet [69] or COCO [55]. This metric takes into account all predictions, offering a solid comparative standard between networks and applications. Once the AP is obtained for each class, a *mean Average Precision* (mAP) for all classes is computed.

Following, a threshold sweep over the prediction confidence from 0% to 100% in 1% steps was performed ($C_{thr1}$). For each step, the predictions with an associated confidence level lower than the $C_{thr1}$ were removed; and the *Precision* and *Recall* metrics from the TP, FP and FN values were calculated.

*Precision* represents the percentage of TP predictions with respect to all predictions (Equation (4)). *Recall* refers to the percentage of TP predictions with respect to all real instances present in the ground-truth data (Equation (5)).

(4) $$Precision=\frac{TP}{TP+FP},$$

(5) $$Recall=\frac{TP}{TP+FN}.$$

Finally, the *F*1 *score* [70] is calculated for each sweep step from its corresponding *Precision* and *Recall* values (Equation (6)). The *F*1 *score* is a measure of overall accuracy. The $C_{thr1}$ associated to the step with the highest *F*1 *score* is selected as the optimal $C_{thr1}$, obtaining the best *Precision* and *Recall* metrics.

(6) $$F1score=2\times \frac{Recall\times Precision}{Recall+Precision}.$$

**Figure 6:**
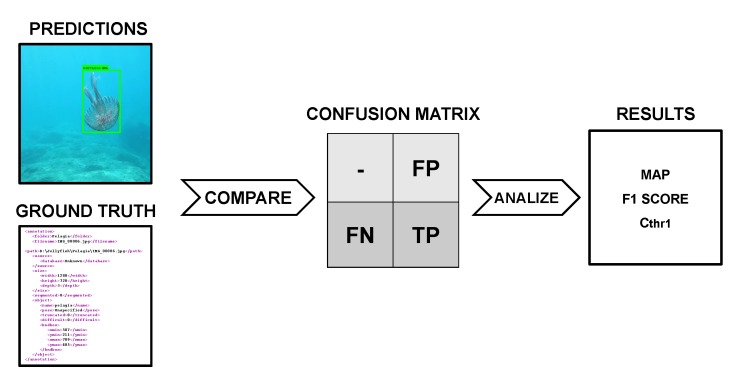
Model evaluation process. The final predictions are compared to their corresponding ground truth using the Intersection over Union (IoU) method and obtaining the False Positive (FP), False Negative (FN) and True Positive (TP) values. From these, the *F*1 *score* at the optimal threshold $C_{thr1}$, altogether with the mean Average Precision (mAP) values are calculated.

Since the main aim in our application is to detect and count the number of jellyfish, finding the optimal $C_{thr1}$ is critical as we need a good trade-off between maximizing the prediction of jellyfish (TP) while minimizing the number of wrongly detected jellyfish (FP). The process that evaluated the prediction performance of the model is represented in Figure 6.

### 4.6. Real-Time Quantification

After training the network and selecting the best hyperparameter values and combination, we assessed the capability of the network at real-time jellyfish monitoring tasks. To do so, a video sequence was manually labeled, indicating the number and classes of jellyfish present at each frame. Subsequently, the same video was analyzed by the neural network. Each time that the network was able to analyze a frame for the video sequence, it generated a predicted information point, containing the number and classes of jellyfish present at the analyzed frame.

The neural network detection may be affected by sporadic changes in luminosity, strange jellyfish positions, water reflexes, etc., resulting in the loss of TP detection or the appearance of FN detection. To minimize the effect of sporadic changes in the detection and improve the quantification performance of the neural network, we implemented diverse time series processing techniques.

Firstly, we performed a window analysis over the predicted information points. This technique takes the information of $W_{size}$ number of predicted information points and processes it to generate a resulting information point (R_I_point). In our case, the value of the resulting information point was taken as the most occurring value from the analyzed window predicted information points. The application of this technique helps to eliminate sporadic detection errors. Three different window sizes were tested: $W_{size}=4,8,12$ information points.

**Figure 7:**
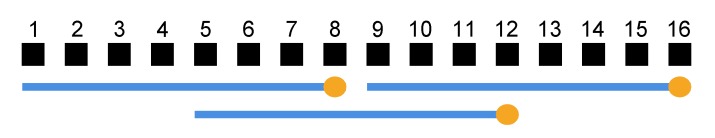
Representation of the window analysis and overlapping techniques ($W_{size}=8$, $W_{over}=50\%$). The black squares represent the predicted information points, the blue lines represent the windows and the orange dots are the resulting information point of each window.

Secondly, we decided to apply an overlap between the information windows in order to preserve the significance of the predicted information points in the transition between windows. This overlap allows us to obtain resulting information points more frequently. Three different window overlaps were tested: $W_{over}=25\%,50\%,75\%$. A representation of the application of these time series processing techniques over a series of predicted information points can be seen in Figure 7.

Due to the implementation of these techniques, the optimal confidence threshold to obtain the best *Similarity* is bound to diverge from the previously selected $C_{thr1}$. So, following the procedure explained in Section 4.5, we performed a threshold sweep over the confidence of the video sequence detection. For each threshold, we applied all combinations of windowing parameters. Finally, for each combination, the $C_{thr2}$ that resulted in the best *Similarity* was selected.

The comparison between the manual and network predictions was carried out by computing the *Similarity* between the manual and neural network quantification, expressed as the percentage of correct resulting information points over the total number of resulting information points (Equation (7)). We classify an information point as correct when it correctly indicates the number and classes of jellyfish present in a determined time of the analyzed video.

(7) $$Similarity=\frac{correct\;R\_I\_point}{total\;R\_I\_point}.$$

## 5. Results and Discussion

This section reports the performance obtained for each experiment in the final predictions and discusses the effect of each hyperparameter over it. Also, it exposes the real-time quantification results obtained from analyzing a video sequence and the conclusions that can be extracted from these. Finally, it presents a comparison between the performance of the selected Inception-ResNet V2 architecture versus two of its main competitors in both final predictions and quantification.

### 5.1. Experiment Performance

Average results obtained from the ten models corresponding to each one of the *K* = 1...12 experiments are shown in Table 2.

All experiments showed mAP values in the 93–95% range, reaching a maximum of 95.0% for experiment 12 and a minimum value of 92.9% for experiment 7. The comparison of AP values for the three species shows that *R. pulmo* and *C. tuberculata* have higher AP values than *P. noctiluca*. This might be related to the fact that *R. pulmo* and *C. tuberculata* are bigger specimens and the shape of their bodies remains relatively unchanged while swimming and therefore they might be easier to identify. On the contrary, in *P. noctiluca* the relative position of the tentacles in relation to the main body (umbrella) changes to a greater extent with the movement of the animal, adopting a multitude of shapes, making it more difficult to identify. Regarding the $C_{thr1}$ and *F*1 *score* values, most experiments found the best *F*1 *score* when applying relatively high $C_{thr1}$ values, indicating that most TP detection had high confidence levels. Experiments showed *F1 scores* ranged from of 93% to 95%, reaching a maximum of 95.2% for experiment 12 again.

**Figure 8:**
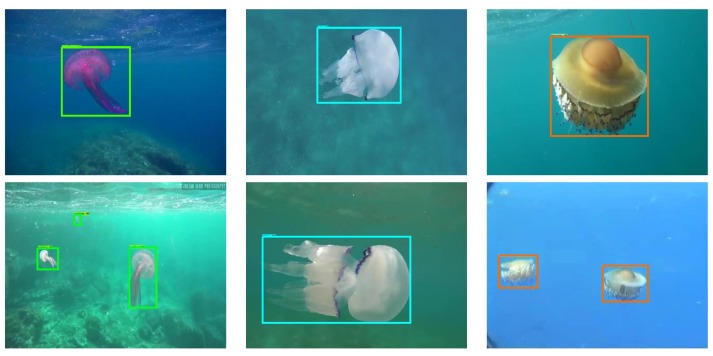
Jellyfish detection examples over test set images. **Left**: green bounding boxes over *P. noctiluca*; **center**: blue boxes over *R. pulmo*; **right**: orange bounding boxes over *C. tuberculata*.

The comparison of the different experiments on a hyperparameter basis indicates that the application of data augmentation, the use of a higher number of iterations and the decay technique application resulted into increased performances. Experiment 12, which featured all three hyperparameters, presented the best performance. Figure 8 illustrates an example of the detection of jellyfish over images from the test set.

### 5.2. Real-Time Quantification

To perform the quantification task and obtain its results, we made use of the best model *M* from experiment 12, containing the previously selected best-performing hyperparameters. We forwarded a 1920×1080 video sequence recorded by the authors using the procedures mentioned in Section 4.2 and analyzed it in real-time. No images from this video had been used either for training nor for testing the network. The duration of the video is approximately 5 min and contains a single jellyfish species (*P. noctiluca*) as, despite the best efforts, no videos with more than one of the studied jellyfish species could be located. This analysis was carried out in a computer with the following specs—processor: Intel i7-7700, RAM: 16 GB, GPU: NVIDIA GeForce GTX 1080).

Table 3 shows the obtained results for all windowing parameter combinations. The third column of the table indicates the time between resulting information points ($T_{R\_I\_point}$) in seconds after applying the time series processing techniques, obtained from Equation (8).

(8) $$T_{R\_I\_point}=\frac{w\_size\times (1-w\_overlap)}{fps},$$

where fps indicates the frame rate at which the network was able to analyze the forwarded video. The Inception ResNet V2 architecture was able to perform the inference of a frame each 0.625 s (1.6 fps).

$T_{R\_I\_point}$ can be adjusted to meet the monitoring target requirements. The $W_{size}$ could be lowered and the $W_{over}$ raised to reduce this time, or the other way around to increase it.

It can be seen that all combinations showed high *Similarity* values, reaching a maximum of 93.8% when using a $W_{size}=12$ predicted information points and an overlapping between windows of $W_{over}=25\%$. Selecting these windowing parameters, a resulting information point is obtained each 5.62 s (following Equation (8)), endorsing that this value is adequate for the monitoring of slow-moving organisms such as jellyfish.

It can also be appreciated that the best *Similarity* for all combinations was achieved when applying much lower $C_{thr2}$ than the $C_{thr1}$ values obtained during the pure prediction task presented in Table 2. The time series processing techniques eliminate spurious FP predictions, allowing us to reduce the $C_{thr2}$ values and introducing low confidence TP predictions while not being punished by low confidence FP.

**Figure 9:**
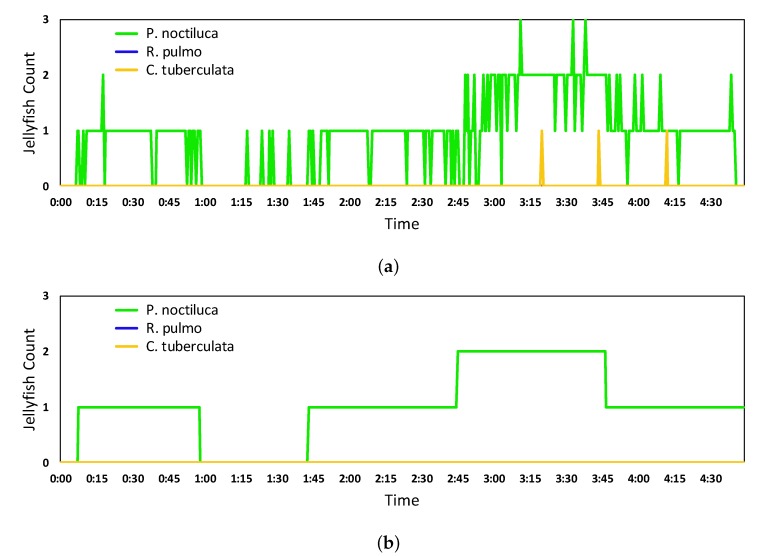
Results of the jellyfish count from Inception ResNet V2 final predictions (**a**) and quantification algorithm (**b**) over a video showcasing nearly 5 min of footage of up to two *P. noctiluca* jellyfish going in and out of the frame.

The solidity of results using the quantification algorithm can be appreciated in Figure 9, which shows the difference between the jellyfish count obtained when using the final predictions versus the application of the quantification algorithm algorithm over the Inception ResNet V2 detection.

Figure 9a shows the count of each studied jellyfish species calculated from the final predictions. It can be seen how this value highly varies in time. Figure 9b shows the count obtained after the quantification algorithm using the windowing parameters that showed the best performance. The count is stable over time and closer to reality.

**Figure 10:**
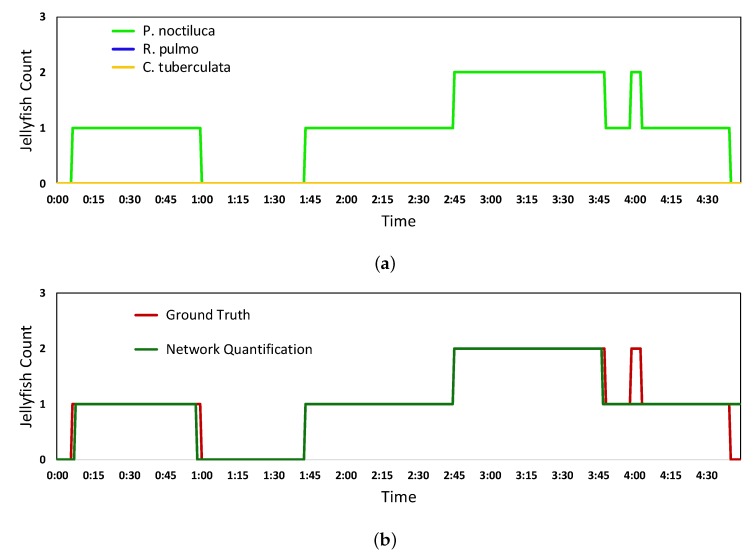
Results of the jellyfish count from manually generated ground truth (**a**) and its comparison against the results from the Inception ResNet V2 network quantification algorithm (**b**).

Additionally, the manually generated jellyfish count, acting as ground truth, for the same video is presented in Figure 10a along with its comparison against the obtained quantification in Figure 10b. The comparison has been made only for the *Pelagia noctiluca* species, as it was the only species present in the video sequence, thus, there is no quantification of errors for the other two species.

Figure 10 shows that some of the divergences can be found when the jellyfish count changes, where the network quantification shows a slower reaction compared to the manual quantification, caused by the computational time of the network and the $T_{R\_I\_point}$ introduced by the time series processing techniques. Also, some other quantification error are due to some timely close resulting information points containing detection errors.

An illustrative video of Jellytoring analyzing the studied video sequence can be seen on the SRV research group web page [71].

### 5.3. Neural Network Performance Comparison

To evaluate the effectiveness of the selected neural network and address its adequacy to our application in terms of detection performance and computational cost, we performed a comparison between the Inception-ResNet V2 architecture and two other object detection architectures, the InceptionV2 [72] and the ResNet101 [58].

These architectures were selected as they are close competitors to Inception-ResNet V2 in terms of detection performance and computational cost trade-off [54].

First, the three architectures were trained and tested over the dataset presented in Section 4.2 with the selected best hyperparameters from Section 5.1 and the 10k-fold cross-validation strategy. The detection performance comparison was conducted using the mAP and *F*1 *score* evaluation metrics. Table 4 shows the comparison between detection performance metrics.

The mAp and *F*1 *score* comparison among the three architectures indicates that Inception-ResNet V2 offers the highest detection performance. ResNet101 architecture shows detection metrics close to those of Inception-ResNet V2 albeit slightly lower. Conversely, Inception V2 shows worse mAP and *F*1 *score* values.

Following, the video sequence presented in Section 5.2 was forwarded into the three architectures and their detection’s were processed by the quantification algorithm.

Table 5 exposes the comparison between quantification results. The presented *Similarity* results are from the best $C_{thr2}$ for each combination. The $W_{size}$ values were adjusted, taking into account each network fps, to maintain the same time between resulting information points as the ones obtained in Table 3.

In terms of fps achieved, the Inception V2 architecture was able to analyze 25.2 frames per second, while the ResNet101 managed to process 10 frames per second. Both architectures achieve higher fps values than the Inception-ResNet V2 architecture (1.6), meaning that higher $W_{size}$ values can be used to incorporate more predicted information points in each window, helping to reduce spurious detection errors. Nevertheless, it can be seen that neither the Inception V2 nor the ResNet101 architectures were able to obtain higher *Similarity* values than the Inception ResNet V2, reaching 73.3% and 90.0%, respectively.

**Figure 11:**
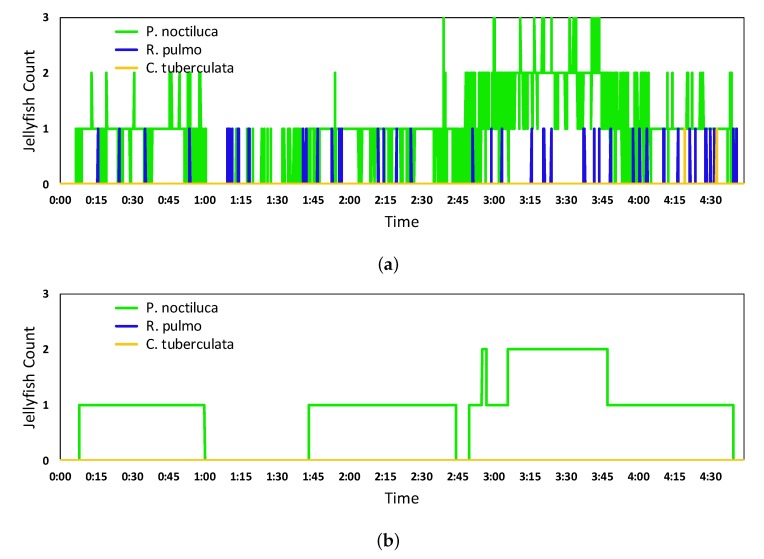
Results of the jellyfish count from the ResNet101 network final predictions (**a**) and quantification algorithm (**b**).

**Figure 12:**
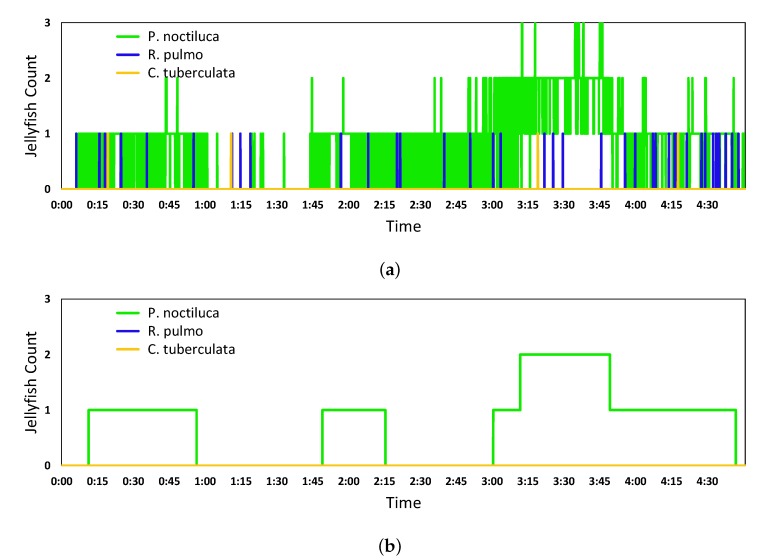
Results of the jellyfish count from the Inception V2 network final predictions (**a**) and quantification algorithm (**b**).

Figure 11a and Figure 12a show the results of the jellyfish count from the ResNet101 and Inception V2 network final predictions, respectively. In the same way, Figure 11b and Figure 12b present the corresponding network quantification when using the best windowing parameters.

The high detection and quantification metrics shown by the Inception-ResNet V2 network make it the most suitable for jellyfish monitoring. The ResNet101 architecture offers a moderate trade-off between computational cost and quantification performance, still reaching good detection and quantification metrics at higher frames per second, making it suitable for detecting and quantifying faster species. The Inception V2 architecture offers a more extreme trade-off between computation cost and quantification performance, providing much lower inference time at still reasonably good detection and quantification metrics.

## 6. Conclusions and Future Work

This paper presents Jellytoring, a system for real-time jellyfish monitoring from underwater video recordings. Jellytoring uses a deep object detection neural network to detect and classify jellyfish instances, combined with a quantification algorithm. A main advantage of this system is that it is able to automatically monitor jellyfish presence without the need for any human interaction, allowing us to generate continuous and precise records. Additionally, the information can be fed to the system in real-time, generating live records.

The neural network evaluation presented very high metrics in the prediction task, reaching a maximum *F*1 *score* of 95.2% when the data augmentation and learning rate decay techniques were applied and the network was trained for 40,000 iterations. On the same page, the best quantification results were obtained when choosing a $W_{size}$ of 12 information points and a $W_{over}$ of 25%, being able to analyze a video sequence with a *Similarity* of 93.8% between the manually generated ground truth and the output of the quantification algorithm. These results indicate that the presented system is able to detect, quantify and monitor jellyfish with high accuracy, thanks to the quantification algorithm that improves the neural network detection.

Additionally, Jellytoring can be customized, widening the applicability of the system. This can be done either by using other network architectures or changing the windowing parameters from the time series processing techniques. Some other possible applications could be the monitoring of other jellyfish species, faster species such as fish, or even other objects like marine waste.

Further developments will focus on lightening the system computational load while maintaining high accuracy levels. Also, we will work on increasing the number of jellyfish species the network can distinguish, widening its spatial application. Our final goal is to implement this system on a floating station and be executed online to monitor the presence and class of jellyfish and relate it to determined water conditions.

We provide our dataset and code, along with the best trained inference frozen model in a GitHub repository [73].

## Figures and Tables

**Table 1:** Hyperparameter values and combinations.

1|c|Index	Data Aug.	Learn. Rate	Iterations
1	No	5 × $10^{-4}$	10k
2			20k
3			40k
4		decay	10k
5			20k
6			40k
7	Yes	5 × $10^{-4}$	10k
8			20k
9			40k
10		decay	10k
11			20k
12			40k

**Table 2:** Results obtained from the evaluation of each experiment *K*, indicating the hyperparameters used along with the AP obtained for each class, the mAP value, optimal $C_{thr1}$ and corresponding *F*1 *score*. Bold data indicates the highest score obtained for each metric.

Exp.	D. aug.	Lr.	Iter.	AP			mAP	$C_{\mathrm{thr}1}$	F1 Score
				P. noct.	R. pulmo	C. tuber.			
1	No	0.0005	10k	85.3%	98.2%	97.2%	93.6%	85%	93.7%
2			20k	86.3%	97.7%	97.3%	93.8%	85%	94.0%
3			40k	86.1%	97.6%	97.1%	93.6%	93%	94.1%
4		decay	10k	86.5%	98.1%	97.5%	94.0%	82%	94.1%
5			20k	86.3%	98.4%	97.3%	94.0%	95%	94.2%
6			40k	85.8%	98.9%	96.6%	93.8%	91%	94.2%
7	Yes	0.0005	10k	84.4%	97.5%	96.7%	92.9%	79%	93.6%
8			20k	86.5%	98.8%	96.7%	94.0%	91%	94.5%
9			40k	86.8%	**99.0%**	96.5%	94.1%	89%	94.8%
10		decay	10k	87.1%	98.5%	96.9%	94.1%	69%	94.6%
11			20k	87.6%	**99.0%**	97.5%	94.7%	86%	95.0%
12			40k	**88.2%**	**99.0%**	**97.7%**	**95.0%**	90%	**95.2%**

**Table 3:** Quantification results obtained from analyzing a video sequence for all windowing parameter combinations. Bold data indicates the highest score obtained for each metric.

$W_{\mathrm{size}}$	$W_{\mathrm{over}}$	$T_{R\_I\_\mathrm{point}}$	$C_{\mathrm{thr}2}$	*Similarity*
4	25%	1.87	11%	87.7%
	50%	1.25	12%	87.9%
	75%	0.62	20%	87.5%
8	25%	3.75	36%	90.5%
	50%	2.50	36%	92.2%
	75%	1.25	36%	90.5%
12	25%	5.62	20%	**93.8%**
	50%	3.75	27%	92.7%
	75%	1.87	27%	92.1%

**Table 4:** Summary of detection performance metrics of Inception-ResNet V2, Inception V2 and ResNet101 neural network architectures. Bold data indicates the highest score obtained for each metric.

Architecture	mAP	F1 Score
Inception V2	76.5%	80.2%
ResNet	93.9%	94.2%
Incep.-ResNet V2	**95.2%**	**95.2%**

**Table 5:** Quantification results of Inception-ResNet V2, Inception V2 and ResNet101 neural network architectures. Bold data indicates the highest score obtained for each metric.

Inception-V2 fps achieved: 25.2				ResNet101 fps achieved: 10.0				Inception-ResNet V2 fps achieved: 1.6		
$W_{\mathrm{size}}$	$W_{\mathrm{over}}$	***Similarity***		$W_{\mathrm{size}}$	$W_{\mathrm{over}}$	***Similarity***		$W_{\mathrm{size}}$	$W_{\mathrm{over}}$	***Similarity***
63	25%	70.3%		25	25%	**90.0%**		4	25%	87.7%
	50%	70.7%			50%	89.3%			50%	87.9%
	75%	72.1%			75%	89.0%			75%	87.5%
126	25%	70.0%		50	25%	87.8%		8	25%	90.5
	50%	72.0%			50%	87.5%			50%	92.2%
	75%	71.3%			75%	86.7%			75%	90.5%
189	25%	69.9%		75	25%	87.8%		12	25%	**93.8%**
	50%	**73.3%**			50%	86.3%			50%	92.7%
	75%	70.9%			75%	84.8%			75%	92.1%
